# Development of an Adverse Drug Reaction Risk Assessment Score among Hospitalized Patients with Chronic Kidney Disease

**DOI:** 10.1371/journal.pone.0095991

**Published:** 2014-04-22

**Authors:** Fatemeh Saheb Sharif-Askari, Syed Azhar Syed Sulaiman, Narjes Saheb Sharif-Askari, Ali Al Sayed Hussain

**Affiliations:** 1 Discipline of Clinical Pharmacy, School of Pharmaceutical Science, Universiti Sains Malaysia, Penang, Malaysia; 2 Pharmacy Department, Dubai Health Authority, Dubai, United Arab Emirates; University of Florence, Italy

## Abstract

**Background:**

Adverse drug reactions (ADRs) represent a major burden on the healthcare system. Chronic kidney disease (CKD) patients are particularly vulnerable to ADRs because they are usually on multiple drug regimens, have multiple comorbidities, and because of alteration in their pharmacokinetics and pharmacodynamic parameters. Therefore, one step towards reducing this burden is to identify patients who are at increased risk of an ADR.

**Objective:**

To develop a method of identifying CKD patients who are at increased risk for experiencing ADRs during hospitalisation.

**Materials and Methods:**

Factors associated with ADRs were identified by using demographic, clinical and laboratory variables of patients with CKD stages 3 to 5 (estimated glomerular filtration rate, 10–59 ml/min/1.73 m^2^) who were admitted between January 1, 2012, and December 31, 2012, to the renal unit of Dubai Hospital. An ADR risk score was developed by constructing a series of logistic regression models. The overall model performance for sequential models was evaluated using Akaike Information Criterion for goodness of fit. Odd ratios of the variables retained in the best model were used to compute the risk scores.

**Results:**

Of 512 patients (mean [SD] age, 60 [16] years), 62 (12.1%) experienced an ADR during their hospitalisation. An ADR risk score included age 65 years or more, female sex, conservatively managed end-stage renal disease, vascular disease, serum level of C-reactive protein more than 10 mg/L, serum level of albumin less than 3.5 g/dL, and the use of 8 medications or more during hospitalization. The C statistic, which assesses the ability of the risk score to predict ADRs, was 0.838; 95% CI, 0.784–0.892).

**Conclusion:**

A score using routinely available patient data can be used to identify CKD patients who are at increased risk of ADRs.

## Introduction

Adverse drug reactions (ADRs) represent a major burden on the healthcare system, and are a common cause of hospital admission as well as of in-hospital morbidity and mortality [1]–[3]. Chronic kidney disease (CKD) patients are particularly vulnerable to ADRs because they are usually on multiple drug regimens, have different comorbid conditions [4], and because of alteration in their pharmacokinetics and pharmacodynamic parameters [5]. Therefore, one important step towards reducing ADRs is to identify those patients who are at increased risk of an ADR and to address their individual risk factors.

Differences in individual genome can be considered as predictors for an ADR [6], however high cost and lack of laboratory facilities limit their applicability in daily clinical practice. Another method is to develop a clinical tool or a risk assessment scale for identifying high-risk patients. Some risk factors for ADRs that have been suggested to date include age, gender, number of drugs the patient is receiving, alcohol intake, comorbidity, and factors that alter drug distribution or metabolism, such as renal or hepatic insufficiency, heart failure and anemia [7]–[13].

Although renal insufficiency is found to be a potential risk factor for ADRs in previous studies [9], [14], [15], there are no methods for identifying and stratifying CKD patients regarding their likelihood of developing an ADR. Hence, based on these considerations, the main aim of this study was to develop a comprehensive and easy applicable method of identification of hospitalized adult patients with CKD stages 3 to 5 who are at increased risk of developing an ADR. The aim was also to create a risk score by using routinely obtained data from hospitalized patients with CKD that can be easily applied in clinical practice.

## Materials and Methods

### Study Design and Participants

This was a prospective, observational study conducted at the renal unit of Dubai Hospital in the United Arab Emirates. Consecutive adult patients with CKD stages 3 to 5 (estimated glomerular filtration rate [eGFR], 10–59 ml/min/1.73 m^2^) who were admitted to the renal unit, between January 1, 2012, and December 31, 2012 were included. Exclusion criteria were: patients aged less than 18 years; patients who were admitted with acute renal failure; and patients who were discharged within 24 hours of admission. Prior to data collection, ethics approval was obtained from the Medical Research Committee of Dubai Health Authority, reference number MRC-SR-10/2011-01. The Medical Research Committee did not required a written informed consent from each study participant, however in case further information were required regarding identification or assessment of each ADR, a verbal informed consent was taken from the respective patient.

### Variables

#### Independent Variables

The principal researcher (F.S.A) used a standardized form to collect patients data at their admission. Patients independent variables included demographic characteristics, such as age and sex; physical examination results, such as blood pressure and weight; comorbid conditions, such as diabetes, hypertension, vascular disease, heart failure, atrial fibrillation and anemia; laboratory tests, such as serum and biochemical parameters; and drugs taken during hospital stay. Drugs were coded according to the Anatomical and Therapeutic Chemical (ATC) classification [16], and categorized with a cutoff of 8. Diagnoses were coded according to the International Classification of Diseases, Tenth Revision, Clinical Modification, codes [17]. Patients comorbid conditions were categorized as present or absent at the time of admission. The estimated glomerular filtration rate (eGFR) was computed using the Modification of Diet and Renal Disease (MDRD) equation [18]:

170 × (Serum Creatinine) ^−0.999^× (Age) ^−0.176^× (Serum Urea Nitrogen) ^−0.170^× (Serum Albumin) ^0.318^

For females, the result was multiplied by 0.762. Based on National Kidney Foundation (NKF), patients with eGFR 30 to 59 ml/min/1.73 m^2^ were at stage 3, those with eGFR 15 to 29 ml/min/1.73 m^2^ were at stage 4, and those with eGFR <15 ml/min/1.73 m^2^ were at (ESRD) end-stage renal disease. Based on the type of renal replacement therapy, patients with ESRD were divided further into the hemodialysis, peritoneal dialysis and conservative management groups [19].

#### Dependent Variables

The outcome of interest was the occurrence of ADR, which was defined according to the Edwards and Aronson [20] definition as: “Any appreciably harmful or unpleasant reaction, resulting from the use of a medicinal product, which predicts hazards from future administration and warrants prevention or specific treatment, or alteration of the dosage regimen, or withdrawal of the product”. Only ADRs that developed during hospital stay were included, while ADRs that caused hospital admission were excluded. All ADRs were identified based on the reported evidences of adverse events in either previously published studies [21], [22] and/or the British National Formulary [23]. For each suspected ADR, detailed information about; causative drug(s), such as administered dosage and frequency; objective data, such as physical examination and laboratory results; subjective data, such as dizziness and rash were collected by principal researcher. The drug related causality was assessed by using Naranjo algorithm [24]. ADRs were classified into definite (score, 9–12 points), probable (score, 5–8 points), possible (score, 1–4 points), or doubtful (score, 0 point). Only definite and probable ADRs taking place during hospital stay were considered for this study. All suspected ADRs were reviewed by a second independent reviewer (N.S.A). In cases where the two reviewers disagreed in the scoring of any ADR, they met to reach an agreement, or the case was referred to a senior researcher for review.

### Statistical Analysis

For the purpose of descriptive analysis, patient’s demographic data, physical examination results, comorbid conditions, laboratory tests and medication usage data were compared in according to the presence of ADRs. We used the t-test or Mann-Whitney U test, depending on the skewness of data, for continuously distributed variables and the chi-square (x^2^) test for categorical variables. Continuous variables were first analyzed without categorization, but a different cutoff value was used in multivariate analysis.

#### Model Development

To meet the objective of the study, a sequential series of logistic regression models was developed using ADR as the dependent variable. We used a combination of medical literature guidance and forward selection method to determine variable selection [25]. At the univariate analysis, variables with a probability value of P<0.05 were entered in the multivariate logistic regression analysis. To prevent model over fitting, the maximum number of variables entered in the multivariate regression models was one variable for every eight ADR events [26]. All selected variables were tested for multicollinearity to avoid any strong correlation between the variables. The presence of collinearity was examined by the evaluation of variance inflation factors and magnitude of standard errors. Variables with more than 10% missing values were not included in the analysis. All other missing data were imputed using the multiple imputation technique with 5 imputations.

Models were developed in accordance with the chronology in which patient data are available in clinical practice. Models were consecutively extended with data from patient demographic, physical examination, comorbid conditions, laboratory test and medications used during hospital stay. In each model, variables with probability value of P>0.10 were excluded from further analysis. All models were adjusted for age, sex and estimated GFR. Results from the multivariate logistic regression were expressed in terms of the odd ratio for a particular variable with accompanying 95% confidence interval.

#### Model Performance

Improvement in model performance was tested using measures for calibration and discrimination. For each model, the calibration was measured using the Hosmer and Lemeshow goodness of fit test [27]. Calibration determines the differences between observed and predicted outcomes for groups of patients, with better model having smaller differences between predicted and observed outcomes [28]. The discriminatory power of each model was assessed using the concordance statistics (C statistics). Discrimination refers to the ability of a model to clearly distinguish between 2 groups of outcomes (discriminate between those patients with and those patients without the risk of developing an ADR) and can range from 0.5 (no discrimination) to 1.0 (perfect discrimination) [29], [30]. The overall model fit for sequential models was compared using the Akaike Information Criterion (AIC), which takes into account both the statistical goodness of fit and the number of variables required to achieve this particular degree of fit, by imposing a penalty for increasing the number of variables. The optimal fitted model was selected by the minimum value of AIC [30].

#### Model Validation

In our study, bootstrapping was used to assess the internal validation of the model [31], [32]. Bootstrapping is a resampling process that enable one to make conclusions about the population that the data originated from by drawing with replacement from the original data set [30]. We drew 1000 bootstrap resamples to evaluate the reliability of the regression coefficients. The standard errors were used to calculate 95% bootstrap confidence interval of odd ratios.

All statistical analysis were performed using SPSS (version 21; SPSS, Inc., Chicago, IL) and STATA 12 statistical software (StataCorp, College station, TX). Two-sided *P* values of less than 0.05 were considered statistically significant.

#### Development of an ADR Risk Score

To obtain an easily applicable prediction rule, odd ratios of the variables retained in the best model were used to compute the risk scores. For example, a point of 1 was given to variables associated with an ADR with an odd ratio between 1.00 and 1.99; a point of 2, to those with an odd ratio between 2.00 and 2.99; a point of 3, to those with an odd ratio between 3.00 and 3.99; a point of 4, to those with an odd ratio between 4.00 and 4.99. For each patients, the score was calculated by an arithmetic sum of points for the variables present. Patients were classified into various cutoff points according to their risk score. Differences in the event rate for increasing ADR risk score values were assessed using the chi-square test for trend [33]. Finally, the predictive ability of the score was evaluated by using the C statistic.

## Results

**Figure 1:**
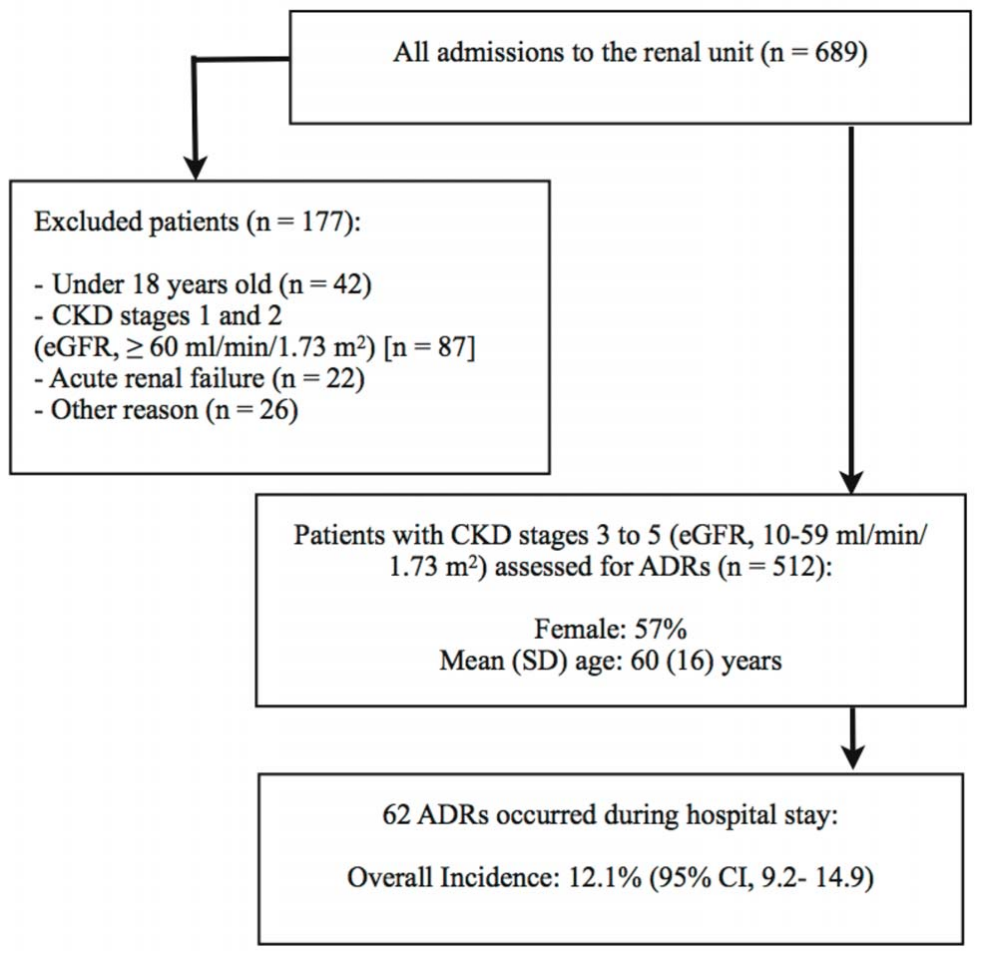
The flow chart of the study. Abbreviations:ADR, adverse drug reaction; CKD, chronic kidney disease; eGFR, estimated glomerular filtration rate; SD, standard deviation; CI, confidence interval.

During the study period, a total of 512 patients with CKD stages 3 to 5 (eGFR, 10–59 ml/min/1.73 m^2^) were admitted to the renal unit. The mean (SD) age of the participants was 60 (16) years, and 57% of patients were female. Out of 512 patients, 62 (12.1%) experienced a probable or definite ADR during hospital stay (Figure 1). The majority of ADRs were caused by anticoagulant drugs (n = 44; 70% of all ADRs), with heparin (n = 18; 28%), enoxaparin (n = 16; 26%) and warfarin (n = 8; 13%) were the most implicated medications (Table 1).

**Table 1:** Medication most commonly implicated in causing the ADRs.

Drug category	No. (%)^a^	Implicated medicines (n)	Adverse Reaction
**Cardiovascular agents**			
	2 (3)	ACEI/ARB	Hyperkalemia
	2 (3)	Furosemide	Hypokalemia
	1 (2)	Propranolol	Dermatitis
**Analgesics**			
	1 (2)	Acetaminophen	Vasovagal attack
**Psychotropics**			
	2 (3)	Benzodiazepine	Drawziness
**Anti-diabetic agents**			
	4 (6)	Sulfonylurea	Hypoglycemia
**Anticoagulants**			
	8 (13)	Warfarin	Bleeding
	16 (26)	Enoxaparin	Bleeding
	11 (17)	Heparin	Bleeding
	7 (11)	Heparin	Thrombocytopenia
	2 (3)	Foundaparinux	Bleeding
**Antithrombotics**			
	1 (2)	Alteplase	Skin bruits
**Antiplatelets**			
	3 (4)	Antiplatelets	Bleeding
**Others**			
	1 (2)	Mycophenolate moftil	Bone marrow suppression
	1 (2)	Darboepoetin	Gastrointestinal disorders

Abbreviations: ACEIs/ARBs, Angiotensin-converting enzyme inhibitors/angiotensin receptor blockers.

a The total number of definite or probable ADRs, n = 62.

### Univariate Analysis

The main characteristics of patients grouped according to the occurrence of ADRs are reported in Table 2. Compared with patients without ADR, those with an ADR were older, with the mean (SD) age of 65 (14) years in the ADR group, compered with 60 (16) years in the non-ADR group (P = 0.023); had more comorbid conditions, with the prevalence of vascular disease (P<0.001); were among conservatively managed ESRD patients (P = 0.002); and were prescribed more medications during hospital stay, with the mean (SD) of 11 (2) medications for patient with an ADR, compared to 7 (2) medications for non-ADR patients (P<0.001). Moreover, patients with an ADR were more hypoalbuminemic, with the mean (SD) serum level of albumin of 3.17 (0.81) g/dL in an ADR group, versus 3.56 (0.62) g/dL in non-ADR group (P<0.001); and had higher serum level of C-Reactive protein (CRP), with the median (IQR) serum level of CRP of 53.50 (134) mg/dL in an ADR group, versus 22.50 (94.75) g/dL in non-ADR group (P<0.001).

**Table 2:** Comparison of Patients Characteristics According to the Occurrence of an ADR.

	No. (%) of Participants		
Characteristics	No ADR (n = 450)	ADR (n = 62)	P Value
**Demographics**			
Age, mean (SD), y	60 (16)	65 (14)	0.023
Female sex	188 (42)	32 (52)	0.142
Male sex	262 (58)	30 (48)	0.142
Current or previous smoking	99 (22)	20 (32)	0.073
**Physical examination, mean (SD)**			
Systolic BP, mm Hg	142 (33)	128 (37)	0.065
Diastolic BP, mm Hg	75 (21)	71 (23)	0.215
**Comorbid conditions**			
Diabetes	324 (72)	51 (82)	0.087
Vascular disease^a^	187 (42)	42 (68)	<0.001
Heart failure	92 (20)	16 (26)	0.332
Atrial fibrillation	34 (8)	9 (14)	0.064
Anemia	209 (46)	34 (55)	0.215
History of gastrointestinal bleeding	64 (14)	14 (23)	0.086
**Renal replacement therapy**			
Hemodialysis	205 (46)	21 (34)	0.082
Peritoneal dialysis	30 (7)	2 (3)	0.294
Conservative management	79 (18)	21 (34)	0.002
**Laboratory data**			
GFR, mL/min/1.73 m^2^			
Baseline, median (IQR)	9.00 (14.00)	10.50 (8.50)	0.234
30–59	58 (13)	6 (10)	0.473
15–29	78 (17)	12 (20)	0.695
<15	314 (70)	44 (71)	0.848
Serum creatinine, median (IQR), mg/dL	5.70 (6.70)	3.95 (4.08)	0.016
Hemoglobin, mean (SD), g/dL	10.22 (2.07)	9.55 (1.97)	0.081
Serum albumin, mean (SD), g/dL	3.56 (0.62)	3.17 (0.81)	<0.001
Serum calcium, mean (SD), mg/dL	8.31 (0.96)	8.41 (0.90)	0.416
Serum phosphate, mean (SD), mg/dL	5.53 (2.23)	4.95 (2.56)	0.083
Serum bicarbonate, mean (SD), mEq/L	20.31 (5.13)	18.85 (5.96)	0.071
C-reactive protein, median (IQR), mg/L	22.50 (94.75)	53.50 (134)	<0.001
**Medication use**			
No. of medications, mean (SD)	7 (2)	11 (2)	<0.001

Abbreviations: ADR, adverse drug reaction; BP, blood pressure; GFR, glomerular filtration rate; IQR, interquartile range; SD, standard deviation.

SI conversions: To convert serum creatinine to µmol/L, multiply by 88.4.

a Vascular disease is defined as presence of coronary artery disease or peripheral vascular disease.

### Model Performance

The odd ratios for the variables and values for discrimination and goodness of fit for successive models are shown in Table 3. Model 1, including age, female sex and eGFR, performed poorly (C statistic, 0.587; 95% CI, 0.507–0.669, and AIC 378.49; P<0.001). However, the C statistic and AIC did improve with the inclusion of conservatively managed ESRD and vascular disease in model 2 (0.689; 95% CI, 0.617–0.760, and 363.94; P<0.001), laboratory values in model 3 (0.761; 95% CI, 0.696–0.826, and 344.60; P<0.001) and the use of 8 or more medications in model 4 (0.813; 95% CI, 0.760–0.867, and 323.23; P<0.001). The Hosmer-Lemeshow statistic was 0.874 for the best model (model 4).

**Table 3:** Odd Ratios and Goodness of Fit for Sequential Models of Prediction of an ADR.

	Models			
	1	2	3	4
Variable	Demographics	Demographics + Comorbid conditions	Demographics + Comorbid conditions + Laboratory data	Demographics + Comorbid conditions + Laboratory data + Medication use
GFR, per ml/min/1.73 m^2^	0.99 (0.97–1.01)	0.99 (0.97–1.02)	1.00 (0.97–1.02)	1.00 (0.97–1.03)
Age, ≥65 y	1.90 (1.09–3.31)	1.34 (0.75–2.41)	1.23 (0.67–2.24)	1.16 (0.62–2.17)
Female sex	1.37 (0.80–2.36)	1.45 (0.83–2.52)	1.50 (0.85–2.66)	1.33 (0.73–2.41)
ESRD, Conservative management		2.29 (1.23–4.27)	1.88 (0.98–3.62)	2.39 (1.21–4.74)
Vascular disease^a^		2.73 (1.50–4.97)	2.40 (1.30–4.45)	2.36 (1.25–4.46)
Serum albumin<3.5 g/dL			2.37 (1.31–4.27)	2.24 (1.21–4.14)
>10 mg/L serum C-reactive protein			2.57 (1.44–4.56)	2.41 (1.33–4.37)
≥ 8 No. of medications				4.64 (2.51–8.59)
C statistic^b^	0.587 (0.507–0.669)	0.689 (0.617–760)	0.761 (0.696–0.826)	0.813 (0.760–0.867)
Akaike Information Criterion^b^	378.49	363.94	344.60	323.23
*p* Value	<0.001	<0.001	<0.001	<0.001

Abbreviation: ESRD, end-stage renal disease; GFR, glomerular filtration rate.

Data are presented as odd ratios (95% confidence interval) unless otherwise specified.

a Vascular disease is defined as presence of coronary artery disease or peripheral vascular disease.

b Higher values for C statistic and lower values for Akaike Information Criterion indicate better models.

### Model Validation

Table 4, presents the results of the logistic regression of the best model along with the bootstrap results. The bootstrap procedure did not change significant variables.

**Table 4:** Logistic Regression and Bootstrapping Model for an ADR.

	Logistic Regression			
Variable	SE	OR (95% CI)	Bootstrap SE	Bootstrap (95% BootCI)
GFR, per ml/min/1.73 m^2^	0.014	1.00 (0.97–1.03)	0.014	(0.97–1.03)
Age, ≥65 y	0.317	1.16 (0.62–2.17)	0.338	(0.60–2.26)
Female sex	0.302	1.33 (0.73–2.41)	0.323	(0.70–2.52)
ESRD, Conservative management	0.348	2.39 (1.21–4.74)	0.370	(1.12–5.09)
Vascular disease^a^	0.325	2.36 (1.24–4.46)	0.357	(1.16–4.77)
Serum albumin<3.5 g/dL	0.313	2.24 (1.21–4.14)	0.323	(1.18–4.27)
>10 mg/L serum C-reactive proteins	0.304	2.41 (1.33–4.37)	0.340	(1.26–4.62)
≥ 8 No. of medications	0.314	4.64 (2.51–8.59)	0.342	(2.39–9.01)

Abbreviation: BootCI, bootstrap confidence interval; CI, confidence interval; ESRD, end- stage renal disease; GFR, glomerular filtration rate; SE, standard error; OR, odd ratio.

a Vascular disease is defined as presence of coronary artery disease or peripheral vascular disease.

### Adverse Drug Reaction Risk Score

As shown in Table 5, the use of 8 or more medications was the variable most strongly associated with ADRs, and scored 4 points, followed by the presence of conservatively managed ESRD and vascular disease, each which scored 2 points. Among laboratory data, serum level of CRP more than 10 mg/L and serum level of albumin less than 3.5 g/dL, each scored 2 points. Female sex was given a score of 1 point and patients aged 65 years or more were scored as 1 point. The score was calculated for each patient by assigning points for each of the variables present and then adding these points. In our data, the score ranged from 0 to 15; the mean (SD) was 4.60 (3.40); the median was 4.00; and the C statistic, which assesses the ability of the risk score to predict ADRs, was 0.838 (95% CI, 0.784–0.892). Table 6, shows the incidence of occurrence of ADRs among patients across various score categories.

**Table 5:** Regression Coefficient and Score of Each Variable Included in Predictive Model.

Variable	OR (95% CI)	Score
**Demographics**		
Age, ≥65 y	1.16 (0.62–2.17)	+1
Female sex	1.33 (0.73–2.41)	+1
**Comorbid conditions**		
ESRD, Conservative management	2.39 (1.21–4.74)	+2
Vascular disease^a^	2.36 (1.24–4.46)	+2
**Laboratory data**		
Serum albumin<3.5 g/dL	2.24 (1.21–4.14)	+2
>10 mg/L serum C-reactive proteins	2.41 (1.33–4.37)	+2
**Medication use**		
≥ 8 No. of medications	4.64 (2.51–8.59)	+4

Abbreviation: CI, confidence interval; ESRD, end-stage renal disease; OR, odd ratio.

a Vascular disease is defined as presence of coronary artery disease or peripheral vascular disease.

**Table 6:** Distribution of Patients According to the Risk Score Derived From Model 4.

Risk score	Total^a^	No. patients with ADRs; n (%)	Incidence of patients with No ADRs; n (%)
**0–1**	99	2 (3)	97 (22)
**2–3**	132	2 (3)	130 (29)
**4–5**	103	5 (8)	98 (22)
**6–7**	65	10 (16)	55 (12)
**8–9**	56	14 (23)	42 (9)
≧**10**	57	29 (47)	28 (6)
**Total**	512	62	450

Abbreviations: ADR, adverse drug reaction.

a Total number of patients per score category.

**Figure 2:**
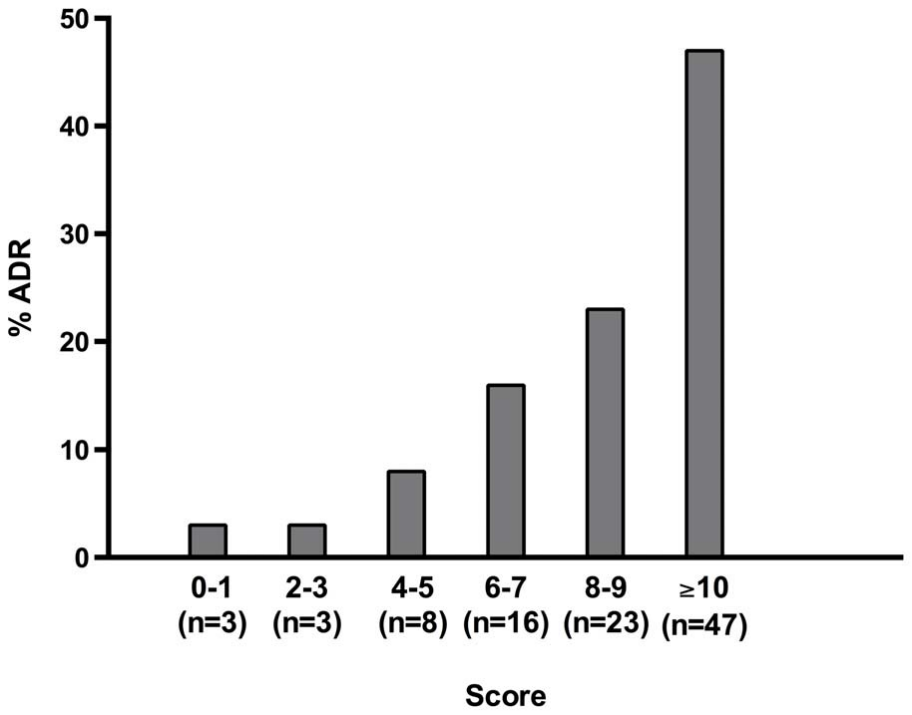
Adverse Drug Reaction (ADR) Rate According to ADR Risk Score Derived From Model 4.

Figure 2, shows that there was a progressive, significant pattern of increasing rate in ADRs as the risk score increased (P<0.001 by chi-square test for trend).

## Discussion

The present study developed a practical and efficient method for identifying CKD patients who are at increased risk for ADR. This method uses patient characteristics data that can be obtained routinely on hospital admission, and that can be incorporated into the clinical practice as a tool to identify CKD patients who are at a high risk of ADRs. Numerous studies have tried to identify and stratify hospitalized patients who are at increased risk for experiencing ADRs [9], [11], [34], however, this study might be the first to incorporate patient laboratory data in the prediction model and thus, this ADR risk score is more representative of every-day clinical practice.

In this study, several risk factors for the development of ADRs in hospitalized patients with CKD were identified. As confirmed by past findings [7], [9], [14], the strongest independent factor was the number of concurrently used medications. Patients with CKD may, of course, have concurrent comorbid conditions that require complex medical regimens [4], and the coadminstration of multiple medications can lead to drug-drug interactions, that increases their possibility of developing ADRs [35].

The current study found that in hospitalized patients with CKD, the rate of developing ADRs increased exponentially with decreasing renal function, with more than two-thirds of ADRs occurring in patients with ESRD. This result is in agreement with a study by Corsonello and colleagues [36] who found an association between patient renal function and the risk of ADRs in elderly hospitalized patients. However, we also found that among ESRD, non-dialysis dependent patients were more than two times more susceptible to develop ADRs compared to hemodialysis patients. This could be because renal impairment related pharmacokinetic and pharmacodynamic changes such as a decrease in the elimination of the parent drug or of toxic metabolites, alteration in drug distribution or protein binding, metabolic abnormalities, or possibly increased target organ susceptibility in uremic patients [5]. This finding emphasizes the need to triage patients for decision regarding the initiation of renal replacement therapy.

In this study, patients with a lower serum level of albumin were at increased risk of experiencing ADRs during hospital stay. This is in line with the Corsonello and colleague [37] study, that showed an association between hypoalbuminemia in patients with concealed renal failure and ADRs. These can also be explained by the decreased binding of albumin-bound drugs, and the accumulation of the unbound fraction in plasma [5].

In addition, patients with vascular disease experienced more ADRs during in hospital stay. Noticeably, patients with CKD are often excluded from coronary artery disease trials; therefore, the safety profile of cardiovascular medications in these patients is mostly based upon knowledge of postmarketing rather than controlled trials [38]. Moreover, in our study, patients who developed ADRs had a higher serum level of CRP, and both CRP value and vascular disease remained significant predictors of developing ADRs when included simultaneously in the regression model. Possible mechanisms include a role for CRP as an inflammatory marker and the formation of atherosclerosis, and its role in the vascular diseases [39].

The ADR risk score has important implications for clinical practice and research. For example, by using this score, lower-risk patients could be managed less extensively, whereas, higher-risk patients could receive more intensive interventions aimed at reducing drug-related adverse outcomes and improving the cost-effectiveness of CKD therapy. Also, by using this score, different risk levels could be used to triage patients for a decision regarding the initiation of renal replacement therapy. Furthermore, the score could be used to identify higher-risk patients who could be enrolled in clinical trials and be evaluated for risk-treatment interactions [40].

The strength of this study is in its development of an ADR risk score for hospitalized patients with moderate to severe CKD. The score is practical because all the variables included can be obtained routinely in a hospital setting. However, the limitation of our study is that the ADR risk score was developed based on hospitalized CKD patients. Thus, it may not be confidently applied in the ambulatory care. Hence, we recommend future studies to evaluate its validity and applicability in ambulatory care.

In conclusion, the current study has developed a practical and efficient risk score for identifying CKD patients who are at increased risk for experiencing ADRs during hospital stay. The score uses routinely available patient data.
